# Action sounds update the mental representation of arm dimension: contributions of kinaesthesia and agency

**DOI:** 10.3389/fpsyg.2015.00689

**Published:** 2015-05-29

**Authors:** Ana Tajadura-Jiménez, Manos Tsakiris, Torsten Marquardt, Nadia Bianchi-Berthouze

**Affiliations:** ^1^UCL Interaction Centre, University College London, University of LondonLondon, UK; ^2^Lab of Action and Body, Department of Psychology, Royal Holloway, University of LondonEgham, UK; ^3^UCL Ear Institute, University College London, University of LondonLondon, UK

**Keywords:** auditory-dependent body-representation, kinaesthesia, agency, action sounds, body-related sensory inputs

## Abstract

Auditory feedback accompanies almost all our actions, but its contribution to body-representation is understudied. Recently it has been shown that the auditory distance of action sounds recalibrates perceived tactile distances on one’s arm, suggesting that action sounds can change the mental representation of arm length. However, the question remains open of what factors play a role in this recalibration. In this study we investigate two of these factors, kinaesthesia, and sense of agency. Across two experiments, we asked participants to tap with their arm on a surface while extending their arm. We manipulated the tapping sounds to originate at double the distance to the tapping locations, as well as their synchrony to the action, which is known to affect feelings of agency over the sounds. Kinaesthetic cues were manipulated by having additional conditions in which participants did not displace their arm but kept tapping either close (Experiment 1) or far (Experiment 2) from their body torso. Results show that both the feelings of agency over the action sounds and kinaesthetic cues signaling arm displacement when displacement of the sound source occurs are necessary to observe changes in perceived tactile distance on the arm. In particular, these cues resulted in the perceived tactile distances on the arm being felt smaller, as compared to distances on a reference location. Moreover, our results provide the first evidence of consciously perceived changes in arm-representation evoked by action sounds and suggest that the observed changes in perceived tactile distance relate to experienced arm elongation. We discuss the observed effects in the context of forward internal models of sensorimotor integration. Our results add to these models by showing that predictions related to action sounds must fit with kinaesthetic cues in order for auditory inputs to change body-representation.

## Introduction

Sounds accompany almost every bodily movement and action we produce. Think for instance about the sound of your footsteps, the impact sound of an object falling from your hand onto the floor, or the sound produced when typing on a keyboard. These sounds are highly rich in information about one’s own body and its effects on the outside world; for instance, footstep sounds vary according to body weight and strength, as well as according to the emotional state of the walker (Li et al., 1991; Bresin et al., 2010; Tajadura-Jiménez et al., 2015a). But, to what extent are we making use of this “soundtrack” that accompanies most of our actions, for gathering information about one’s actions and body? Here we focus on recent findings that sounds produced when tapping one’s hand on a surface recalibrate the mental representation of arm length (Tajadura-Jiménez et al., 2012). We specifically seek to disambiguate the effects of kinaesthetic cues and feelings of agency in this recalibration.

Action- and body-awareness are critical for our interaction with the environment. For instance, according to our perceived body dimensions, we may ponder whether we can reach a particular object or whether there is enough space for us to get onto a crowded bus. Importantly, research has shown that the mental representation of our body (i.e., our body-representation) is not fixed, but it is continuously updated by the body-related multisensory cues received from the environment (de Vignemont, 2010; Longo et al., 2010; Serino and Haggard, 2010). For example, an artificial hand may feel like part of one’s own body when one sees it being touched and in synchrony receives touch on one’s own, unseen, hand. This is the result of the integration of information coming from different sensory channels – vision, touch, and proprioception (Botvinick and Cohen, 1998). Similarly, altering proprioception (de Vignemont et al., 2005; Ehrsson et al., 2005) or vestibular information (Lopez et al., 2012; although see also Ferrè et al., 2013) may result in perceived distortions in body size. Studies using virtual reality set-ups have shown that observing a very long arm (Kilteni et al., 2012; Preston and Newport, 2012) or a very large or very small body (van der Hoort et al., 2011) can result in the illusion of owning that arm or body, provided that visuo-tactile and visuo-motor temporal and spatial congruency is kept constant between the observed body and one’s own felt body. Furthermore, using a tool to act with one’s arm upon relatively distant objects can also result in an increase of represented arm length (Cardinali et al., 2009, 2012; Canzoneri et al., 2013b).

Despite this known link between body-related sensory cues and body-representation, the contribution of auditory cues to body-awareness has been addressed only in a few research studies. It has been shown that action sounds recruit motor areas of the brain that are involved in the planning, preparation, and observation of these actions (Aglioti and Pazzaglia, 2010; see Pazzaglia et al., 2008 for related findings in the visual domain). In addition, there are evidences that self-produced action sounds can also influence the way actions are subsequently performed. For example, altering in real-time the sound of someone’s footsteps influences her walking style (Bresin et al., 2010; Menzer et al., 2010) and altering cues related to applied strength on sounds generated by tapping one’s hand on a surface influences the tapping behavior (Tajadura-Jiménez et al., 2015b). Regarding awareness of one’s own body, on the one hand, it is known that blocking audition by wearing earplugs often results on people reporting an altered body-awareness, apart from a sensation of detachment from the surroundings (Murray et al., 2000). On the other hand, the provision of sound feedback on body movement of a person with reduced body awareness and mobility is known to increase physical self-efficacy (Singh et al., 2014).

In addition to these links between movement and self-produced sound, a few studies have started to show that self-produced sounds contribute to update body-representation. For instance, it has been shown that self-produced sounds update the representation of one’s own entire body size and weight (Tajadura-Jiménez et al., 2015a) and even the experienced material of one’s own body (Senna et al., 2014). The former was achieved by altering the frequency of the self-produced walking sounds, and the latter by altering the sound of the impact of an object on one’s hand. The first demonstration of a link between audition and body-representation was actually provided by a study in which we showed that represented limb length updates by action sounds (Tajadura-Jiménez et al., 2012). In that study we asked participants to tap on a surface while progressively extending their arm sideways. Exposure to tapping sounds originating at double the distance at which participants actually tapped, and presented in synchrony with the taps of participants (Double distance – 2D – condition), changed the perception of tactile distance on the tapping arm, as compared to the perceived tactile distances before tapping. These changes in perceived tactile distances on the arm evidenced a change in represented arm length (e.g., Taylor-Clarke et al., 2004; de Vignemont et al., 2005; Canzoneri et al., 2013a,b). The effects were not observed when the tapping sounds originated at quadruple the distance (4D condition; see also Kilteni et al., 2012, for similar findings on plasticity of represented arm length when manipulating visual cues) or when the sounds were presented in asynchrony with participants’ taps (Double distance asynchronous – 2DA – condition). Self-reports showed that in the 2D condition, as opposed to the 4D condition, participants felt that sound and tap originated at the same location. They also showed that in the 2D condition, as opposed to the 2DA condition, participants felt that the sound was caused by their own hand tapping and that they were in control of their arm. Indeed, temporal contingency is known to be crucial for correct action attribution (Moore et al., 2009). Tajadura-Jiménez et al. (2012) also ran a second experiment in which participants did not generate the taps and did not displace their arm, but they received externally-generated taps to their still arm. Results showed that simply hearing sounds in synchrony and at double the distance at which taps are felt, while keeping the arm stationary, does not elicit changes in perceived tactile distance.

Hence, several factors might be implicated in the auditory-induced changes in perceived tactile distance, namely (1) the magnitude of the manipulation of auditory distance, (2) the synchrony between the tapping sounds and the participants’ taps, (3) the feeling of being the agent of the tapping sounds, and (4) the displacement of the arm when tapping and when displacement of the sound occurs. Which of these factors are necessary and/or sufficient to observe an effect on perceived tactile distance remains unknown. Tajadura-Jiménez et al. (2012) addressed the two first factors and showed that hearing sounds in synchrony and at double the distance at which taps occur were necessary but not sufficient factors to elicit changes in perceived tactile distance. Hence, a remaining question is about the third and fourth factors described above. We hypothesize that a coherent representation of the motor command sent to the tapping arm and the sound feedback received from the tapping action needs to arise during the audio-tactile adaptation in order to observe changes in felt tactile distance. According to the ‘forward internal models’ of the motor system (e.g., Wolpert and Ghahramani, 2000), both temporal and spatial mismatches between motor and sensory representations reduce the likelihood that different sources of sensorimotor information merge to form a coherent and robust percept (Ernst and Bülthoff, 2004), and they interfere with the sense of control or agency over one’s action (Blakemore et al., 2002).

We sought to disambiguate the effect of kinaesthetic cues from the feelings of agency on the observed auditory-driven changes in the representation of arm dimension. For that reason, we opted for keeping the sound presentation equal to that in the study by Tajadura-Jiménez et al. (2012), and manipulated instead kinesthetic cues and the feelings of agency over the generated tapping sounds across different conditions. We asked participants to tap with their arm on a surface. In the “Displacement” conditions participants were required to tap while extending their arm, and they were presented with tapping sounds that originated at double the distance to the tapping locations. Feelings of agency over the tapping sounds were manipulated by presenting the tapping sounds either in synchrony or in asynchrony with the tapping actions (i.e., we expected agency to be preserved only in the synchronous conditions). Across two experiments kinaesthetic cues signaling arm displacement, and therefore change in hand position, were manipulated by having additional control conditions (“No Displacement” conditions) in which participants did not displace their arm but kept tapping at a fixed location, which was either close to their body torso (i.e., arm flexed, in Experiment 1) or far from it (i.e., arm stretched, in Experiment 2). Importantly, the tapping sounds were presented at the same locations across all experimental conditions, with the tapping sounds originating at double the distance to the points where participants tapped during the arm Displacement conditions. Having two posture positions for the No Displacement conditions (i.e., arm flexed in Experiment 1 and arm completely stretched in Experiment 2) allowed controlling for the effect of distance between hand and body torso (close or far) and for the effect of distance between hand and sound source, which was larger for the posture adopted in Experiment 1 than for the posture in Experiment 2. We quantified the effects on subjective feelings and on perceived tactile distance related to represented arm length.

## Experiment 1

### Materials and Methods

#### Participants

Twenty participants (*M*_age_ ± SD = 22.85 ± 2.5 years; age range from 18 to 28 years; 16 females) took part individually in the experiment. The sample size was chosen by a power analysis calculation based on our previous work (Tajadura-Jiménez et al., 2012). This calculation showed that with a sample size of 14, there was 80% likelihood that the study will yield a statistically significant difference between the means of the Synchronous and Asynchronous Displacement conditions. All participants reported having normal hearing and normal tactile perception, and were naïve as to the purposes of the study. They were paid for their time and gave their informed consent prior to their inclusion in the studies. The experiment was conducted in accordance with the ethical standards laid down in the 1964 Declaration of Helsinki and approved by the ethics committee of University College London.

#### Apparatus and Materials

**FIGURE 1:**
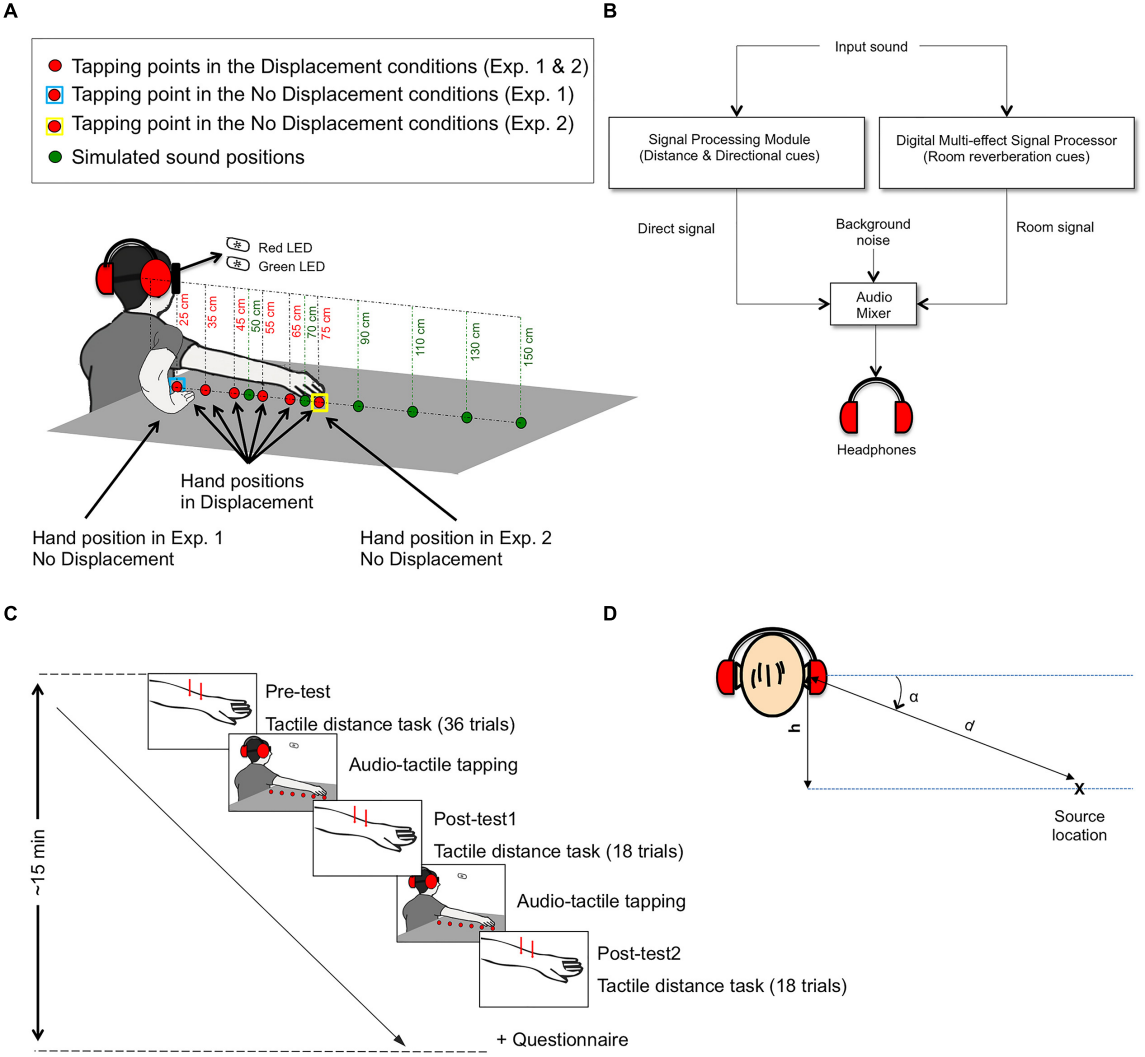
**(A)** Experimental set-up, **(B)** connections of the physical components used for the sound simulation, **(C)** experimental timeline, and **(D)** parameters used for the simulation of auditory distance. In panel A the two right arms displayed correspond, respectively, to the position adopted during the No Displacement condition in Experiment 1 (arm flexed) and the No Displacement condition in Experiment 2 (arm extended). In the Displacement conditions participants started tapping at the first point, with the arm flexed, and then progressively moved their hand along the six tapping-positions ending with the arm extended. **(D)** Illustrates the parameters that were used to calculate the elevation angle (α): height (*h*) between the participants’ right ear and the surface of the table and distance (*d*) from sound source location to ear. The simulation considered the propagation time of sound through the air Δ*t* (which increased with *d*), directional cues and room reverberation (see Materials and Methods).

A schema of the experimental set-up is displayed in **Figure 1A**. Participants were seated in a chair, blindfolded, and wearing a pair of closed headphones with very high passive ambient noise attenuation (Sennheiser HDA 200). A pair of light-emitting diodes (LED), one green and one red, was positioned in front of the participants, at eye level and a distance of 50 cm. They were bright enough so that participants could see the light through the blindfold. The green LED served as the center fixation point, and the red LED was used by participants to perform the experimental task, as described in the next section. During the experimental blocks participants were instructed to refrain from turning their head sideways from the fixation point.

A table was placed to the right of the participants. The height (*h*) between the participants’ right ear and the surface of the table was approximately 40 cm. The participants were instructed to tap on the surface of the table, at six different positions (“tapping-positions”), which were located 90° to the right at 25 cm, 35 cm, 45 cm, 55 cm, 65 cm, and 75 cm from a vertical line traced between the participants’ right ear and the table surface.

We simulated the auditory “source locations” by using virtual acoustic techniques (e.g., Begault, 1994). A “dry” recording of two fingers tapping on a cardboard box was made in an anechoic chamber. The recording lasted 125-ms and had a broad spectrum. This recording was later loaded onto a real-time signal processing module (RP2.1, Tucker–Davis Technologies) to manipulate the virtual location of the sound arriving directly from the sound-source. In a parallel processing path, room reverberation was added to the “dry” signal using a digital multi-effect signal processor (Digitech DSP 128 plus) to simulate a small room with low reflective surfaces (RT60 = 0.36 s). The direct and the room signal were then added (TEAC audio mixer model 2A) and presented to the right and left ear via stereo headphones (**Figure 1B**).

The “dry” pre-recorded sound was modified in the real-time-processor to provide the listener with distance cues and directional cues using RPvdsEx software (Tucker–Davis Technologies). Increased source distance was simulated by increasing delay and decreasing intensity of the direct signal, thus decreasing the direct-to-reverberation ratio, which is one of the strongest distance cues in reverberant environments. The intensity *I* of the direct sound decreased with the square-power of the distance to ear *d* ($I=\frac{1}{d^{2}}$; see **Figure 1D**). A delayΔt, of 3 ms per meter, was introduced to the direct sound to simulate the velocity of sound in air:

$$\Delta t=\frac{d}{speedofsound}=\frac{d[m]}{350[\frac{m}{s}]}=d\times 0.003[s]$$

It should be noted that it is not this delay of the direct sound relative to the tapping sensation, but the consequently decreasing difference between the delay of the direct sound and fixed delay of the first reflection that provides a distance cue. The latency of the signal processing module (RP2) consisted largely of the A/D and D/A conversion time, and was in total less than 4 ms. Such short latency is unperceivable across sensory modalities, as it falls well within the intersensory temporal synchrony window (Lewkowicz, 1996, 1999).

Directional cues were then introduced to the direct sound by convolving the signal with the left and right sets of head-related transfer functions (HRTFs) that correspond to the desired spatial direction of the source. Sets of generic HRTFs are provided by RPvdsEx software. We used the set for 90° azimuth, and an elevation angle α (see **Figure 1D**):

α=arcoosin(h/d)

A piezoelectric transducer (Schaller Oyster 723 Piezo Transducer Pickup), attached to the table, was used to detect the participants’ taps and trigger the auditory stimulation. In the Synchronous condition, the auditory stimulus was presented in synchrony with the participant’s tap on the table. In the Asynchronous condition, the auditory stimulus was presented with a small delay with respect to the participant’s tap. This delay varied randomly over a range of 300–800 ms. It should be noted that the minimum delay value (i.e., 300 ms) was chosen to fall outside of the multisensory integration window during which asynchronous stimuli in different modalities are perceived as simultaneous (Lewkowicz, 1996, 1999).

An array of six spatial “source locations” was simulated. The source locations were aligned with the tapping-positions but were at double the distance than those (i.e., the “source locations” were separated by 20 cm). An additional array (“practice array”) had “source locations” identical to the tapping-positions and was simulated for the practice block that participants performed to get familiar with the tasks.

In the arm Displacement conditions, the tapping sounds originated at double the distance to the tapping-positions (the Synchronous and Asynchronous conditions resemble, therefore, the 2D, and 2DA conditions in Tajadura-Jiménez et al., 2012). Hence, the last auditory stimulus of the experimental trial was delivered from the sixth source location in the array, 150 cm away from the vertical line traced from the participants’ ear (while the last tapping-position was 75 cm away). In the No Displacement conditions, the tapping sounds originated at exactly the same locations than during the arm Displacement conditions. Thus, these conditions did not differ in the sounds presented, but rather in that participants did not displace their arm while tapping in the No Displacement conditions and they did in the Displacement conditions. The actual sound of the participants’ taps on the table was attenuated by the high ambient-noise attenuation headphones, and masked by adding background noise (interaurally uncorrelated pink noise, 20–13000 Hz) to the headphone signals throughout the entire experimental session (see Procedure section).

The stimuli for the tactile distance task consisted of three pairs of wooden posts (diameter 3 mm) mounted in foam board, as in Longo and Haggard’s (2011), study. The pairs of posts differed in the separation between the posts, which was fixed at 4, 5, or 6 cm. They were presented at two different body locations, the participant’s right forearm (test stimuli) and the forehead (reference stimuli; see Canzoneri et al., 2013a,b, for similar procedure). The minimum distance of 4 cm was chosen to be clearly suprathreshold at both body locations (Nolan, 1982, 1985). Each tactile contact lasted for approximately 1 s.

MATLAB software was used to control stimulus delivery and record responses.

#### Audio-Tactile “Tapping” Task

Participants were required to centrally fixate the green LED and to perform the simple action of tapping on the table using their right hand, while keeping the arm ventral side down. They tapped at the first taping-position for ten times and the auditory stimulus was delivered at the first source location in the array, in synchrony or in asynchrony with the participant’s tapping, depending on the condition. Participants were asked to pace their rhythm keeping a frequency of approximately one tap per second.

In the Displacement trials, after ten taps, a signal (red LED) indicated the participants to extend their arm rightward by 10 cm, and tap again for 10 times at the new tapping-position, with the auditory stimulus presented from the subsequent source location in the array (i.e., at double the distance to the tapping-position). This procedure was repeated six times, for a total of 60 taps on the table, in the Displacement trials 10 at each of the six tapping-positions, and in the No Displacement trials 60 taps at the same, first tapping-position. In the No Displacement trials, participants kept tapping at the first tapping-position while the auditory stimulus changed to the subsequent source location in the array every ten taps. After these 60 taps, participants were asked to repeat the procedure again, starting from the first tapping-position. Hence, an experimental trial included two sets of 60 taps. At the end of the Displacement trials, participants were instructed to keep the right hand open on the last tapping-position. At the end of the No Displacement trials, they were instructed to fully extend their arm and place their hand open on the last tapping-position, being assisted by the experimenter.

#### “Tactile Distance” Task

This task, adapted from previous studies (de Vignemont et al., 2005; Lopez et al., 2012; Tajadura-Jiménez et al., 2012; Canzoneri et al., 2013a,b), serves as an indirect measure of the mentally represented body part size. Participants were required to adopt the same position that they had by the end of the audio-tactile “tapping” task, i.e., right arm extended laterally, ventral side up, with the hand open, and placed approximately 75 cm away. Dual tactile stimuli were delivered manually by the experimenter using pairs of wooden posts on two different body locations consequently (right forearm – test location and forehead – reference location), in a randomized order. The duration of each touch was approximately 1 s, with approximately 1 s between the touches to the two body locations. A sequence of 36 tactile trials, which constituted one “tactile distance” block, was generated beforehand and randomized. In one third of the trials the tactile distance on the test and reference locations was the same, in another third differed by ±1 cm and in the last third differed by ±2 cm. The task for participants was to indicate verbally whether the two points felt farther apart in the first or the second stimulated location (adapted from Longo and Haggard, 2011).

#### Procedure

Participants sat on a chair and a sound test was performed to check listeners’ perceived azimuth for the simulated sound sources. This test revealed that participants perceived the sound sources originating on average at 102° (SE = 5.26; range from 60 to 140°), which corresponds to locations on the right, slightly back from participants’ right ear. This test provided evidence that the simulated sound direction was perceived as expected. Then, participants were instructed and were asked to practice the tapping, paying attention to keep the required tapping rhythm (approximately one tap per second) and the location of the six tapping-positions (separated by 10 cm). Participants first practiced without wearing the blindfold, and then, once again wearing the blindfold, with the experimenter giving them feedback on their performance and correcting their movements if necessary. Next, they completed a full experimental practice block (Synchronous – Displacement, as described below) to familiarize themselves with the audio-tactile “tapping” and the tactile distance tasks. The audio-tactile “tapping” task in this practice block differed from the one in the experimental blocks in that the “practice array” of source locations was used. Given this extensive practice before the experiment start, during the experiment participants managed to tap approximately at the tapping-positions. The experimenter kept close to participants and visually monitored that the required pace and distances of movement were kept during the whole experiment, and when necessary, corrected participants by grabbing and leading their hand to the exact tapping-position.

Next, participants completed four experimental blocks, each containing five stages (See **Figure 1C**): pre-stimulation tactile distance task (Pre-test, 36 trials), audio-tactile tapping task, post-stimulation tactile distance task (Post-test, 18 trials), audio-tactile tapping task, post-stimulation tactile distance task (Post-test, 18 trials). The experimental blocks differed in the auditory condition (Synchronous or Asynchronous) for the audio-tactile tapping task and in the arm displacement condition (Displacement or No Displacement). The Pre-test values were taken as baseline measures to which refer the Post-test values. The Post-test was split in two parts (18 trials each), with participants performing a second round of the audio-tactile tapping task in between, to ensure that the effect of the audio-tactile tapping task was not lost due to the length of the procedure of the tactile distance task. Future studies may determine how long the effects of the audio-tactile tapping task last. Participants were blindfolded throughout the experimental block and were not allowed to see the tactile stimuli at any point during the experiment.

At the end of each block, the subjective experience of participants during the audio-tactile tapping task was assessed with a questionnaire containing eight statements, adapted from our previous study (Tajadura-Jiménez et al., 2012). The list of statements is presented in the Subjective Results section. Participants rated their level of agreement with the statements using a 7-item Likert scale, ranging from -3 (strongly disagree) to +3 (strongly agree), with 0 referring to “neither agree, nor disagree.”

The order of presentation of the blocks was randomized, with each experimental block lasting on average for 15 min. This resulted in a total duration of the experiment of about 90 min (four experimental blocks and one practice block, plus instructions and debriefing).

#### Data Analyses

**FIGURE 2:**
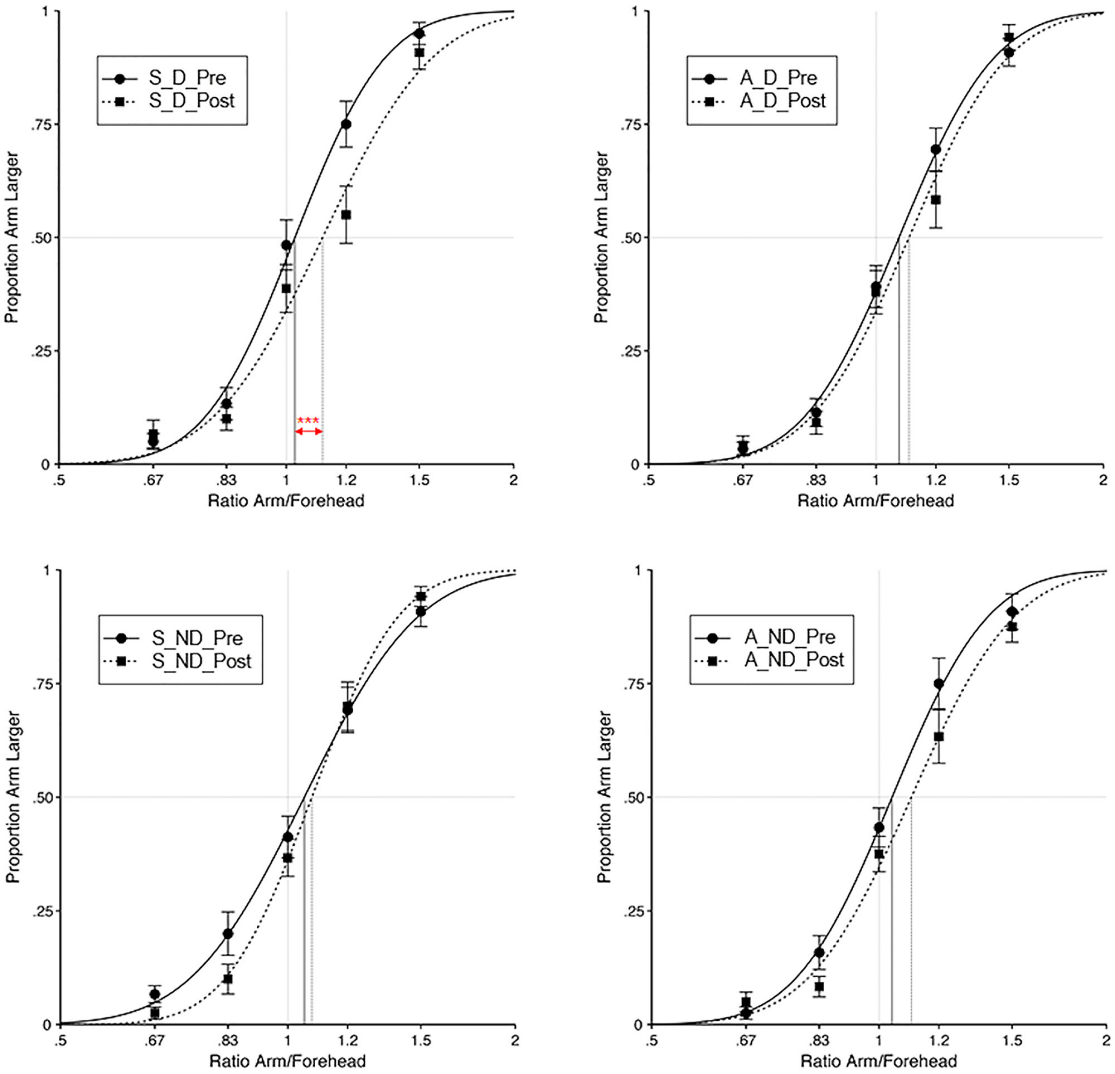
**Results from Experiment 1.** For each experimental condition (S–D, Synchronous – Displacement; S–ND, Synchronous – No Displacement; A–D, Asynchronous – Displacement; A–ND, Asynchronous – No Displacement) and for both the Pre- and Post-test, the proportion of judgements that the distance between dual tactile stimuli on the right arm felt greater than on the forehead was analyzed as a function of the ratio of the length of the arm and forehead stimuli (i.e., 4/6, 5/6, 1, 6/5, or 6/4). Curves are cumulative Gaussian function fits to the group data, for each condition, with least-squared regression. Error bars indicate the SEM. Vertical lines indicate the interpolated points of subjective equality (PSE) between the perceived distance on the arm and on the forehead. Red asterisks denote a significant change in PSE from Pre- to Post-test (^∗∗∗^denotes *p* < 0.001, corrected for multiple comparisons). Note that an increase in the PSE meant that perceived tactile distances on the arm were felt smaller, as compared to distances on a reference location.

For data analyses, we followed the procedure described in Longo and Haggard (2011). For each experimental condition, and for both the Pre- and Post-test, the proportion of judgments that the distance between dual tactile stimuli on the right arm felt greater than on the forehead was analyzed as a function of the ratio of the length of the arm and forehead stimuli (i.e., 4/6, 5/6, 1, 6/5, or 6/4). The proportion of judgments was plotted logarithmically to produce a symmetrical distribution about the point of actual equality (i.e., the point at which the ratio equals 1; see **Figure 2**). Cumulative Gaussian functions were fit to each participant’s data with least-squares regression using R 3.0.1. The point of subjective equality (PSE) was calculated as the point at which the fitted psychometric function crossed 50%. Thus, the PSE corresponds to the ratio of the length of the arm and forehead stimuli for which participants perceived the distance between dual stimuli on both locations to be the same. Given that tactile distance perception is directly related to tactile sensitivity (Taylor-Clarke et al., 2004; Longo and Haggard, 2011), on average a PSE greater than 1 is expected for the Pre-test, given the greater tactile sensitivity of the forehead with respect to the forearm. Given that tactile distance perception also links to the size of the represented body part (Taylor-Clarke et al., 2004; de Vignemont et al., 2005; Longo and Haggard, 2011; Tajadura-Jiménez et al., 2012; Canzoneri et al., 2013a,b), a change in PSE from Pre- to Post-test would provide evidence of the effect of the audio-tactile tapping task on the size of the represented forearm. For all statistical tests alpha level was set at 0.05, 2-tailed.

### Results

#### Behavioral Results

The mean PSE values ± SE are presented in **Table 1**. Initial analyses did not show any difference in the Pre-test PSE values across the different trial conditions (*p* > 0.05), thus confirming the validity of the Pre-test values as baseline. In addition, another initial analysis was performed to investigate potential differences between the two Post-test sets of 18 trials in each condition. A 4 × 2 × 5 analysis of variance (ANOVA) with the factors ‘condition’ (Synchronous – Displacement, Asynchronous – Displacement, Synchronous – No Displacement, Asynchronous – No Displacement), ‘Post-test set’ (first, second), and ‘ratio of the length of the arm and forehead stimuli’ (4/6, 5/6, 1, 6/5, or 6/4) did not show any significant effect or interaction of the factor ‘Post-test set’ (all *p*s > 0.05), thus justifying the treatment of both Post-tests as a single test.

**Table 1:** Results from Experiment 1.

Time of test	Synchronous – Displacement	Asynchronous – Displacement	Synchronous – No Displacement	Asynchronous – No Displacement
Pre-test	1.035 (0.031)	1.085 (0.03)	1.068 (0.037)	1.067 (0.043)
Post-test	1.151 (0.044)	1.123 (0.032)	1.078 (0.026)	1.115 (0.032)

Mean point of subjective equality (PSE ± SE) for each experimental condition and for both the Pre- and Post-test.

Our main analysis focused on the effect of audio-tactile stimulation across conditions. A normality check of the residuals with Shapiro–Wilk tests and Q–Q plots showed moderate deviations from normality for three out of the eight variables (Synchronous – Displacement pre-test: *W*(20) = 0.87; *p* = 0.014; Asynchronous – No Displacement pre-test: *W*(20) = 0.73; *p* < 0.001; Asynchronous – No Displacement post-test: *W*(20) = 0.87; *p* = 0.013). Given that ANOVAs are quite robust to moderate deviations from normality (e.g., McDonald, 2014) we opted for the use of ANOVAs, which allow a factorial design and to explore the interaction between factors. Pre- and Post-test PSE values were submitted to a 2 × 2 × 2 within-subjects ANOVA with ‘audio-tactile synchronicity’ (Synchronous and Asynchronous), ‘arm displacement’ (Displacement and No Displacement) and ‘time of test’ (Pre-test and Post-test) as factors. The 3-way interaction ‘audio-tactile synchronicity’ by ‘arm displacement’ by ‘time of test’ was significant [*F*(1,19) = 4.51; *p* = 0.047], as well as the main effect of ‘time of test’ [*F*(1,19) = 18.39; *p* < 0.001], while the other main effects or interactions failed to reach significance (all *p*s > 0.05).

In order to explore the 3-way interaction, we conducted two further 2 × 2 within-subjects ANOVA, one for the Displacement and one for the No Displacement condition, with factors ‘audio-tactile synchronicity’ (Synchronous and Asynchronous) and ‘time of test’ (Pre-test and Post-test). The ANOVA for the Displacement condition revealed a significant main effect of ‘time of test’ [*F*(1,19) = 15.26; *p* = 0.001], as well as a significant 2-way interaction ‘audio-tactile synchronicity’ by ‘time of test’ [*F*(1,19) = 11.11; *p* = 0.003], while the main effect of ‘audio-tactile synchronicity’ was not significant (*p* > 0.05). Independent-samples *t*-tests showed that the observed interaction was driven by a significant increase in the PSE from Pre- to Post-test in the Synchronous – Displacement condition [*t*(19) = -4.49, *p* < 0.001], which was not observed for the Asynchronous – Displacement condition (*p* > 0.05). Such increase in the PSE meant that exposure to the Synchronous – Displacement condition resulted in the perceived tactile distances on the arm being felt smaller, as compared to distances on a reference location. The ANOVA for the No Displacement condition did not yield any significant main effect or interaction (all *p*s > 0.05).

#### Subjective Results

The full set of statements, mean responses and tests for significance are presented in **Table 2**. In order to investigate the effect of audio-tactile stimulation on the subjective experience of participants across the conditions, first, we tested whether the distributions of the obtained data were normal using the Shapiro–Wilk test. None of the variables passed the normality test, and therefore we used non-parametrical statistical tests to analyze the data (Friedman and Wilcoxon Signed Ranks Test). We observed significant differences between the four conditions for all statements except S4 and S8.

**Table 2:** Mean ratings (±SE) and tests for significance for each questionnaire item across conditions in Experiment 1.

During the audio-tactile stimulation it seemed like…	Mean ratings (±SE) and results of Friedman test	Synchronous vs. Asynchronous (α = 0.025)	Displacement vs. No Displacement (α = 0.025)	S–D vs. A–D (α = 0.017)	S–D vs. S–ND (α = 0.017)	S–D vs. A–ND (α = 0.017)
S1: …the sound I heard was caused by me	**S–D:** 1.9 (0.38) **A–D:** -0.5 (0.5) **S–ND:** 1.15 (0.41) **A–ND:** -1.05 (0.35)	***z* = 3.93** ***p* < 0.001**	***z* = 2.30** ***p* = 0.021**	***z* = 3.34** ***p* = 0.001**	*z* = 1.38 *p* = 0.17	***z* = 3.64** ***p* = 0.000**
	**χ^**2**^(3) = 31.35, *p* < 0.001**					
S2: …my hand was at the same location as the sound	**S–D:** 1.25 (0.45) **A–D:** -0.15 (0.42) **S–ND:** -0.85 (0.45) **A–ND:** -1.5 (0.39)	*z* = 2.08 *p* = 0.037	***z* = 2.88** ***p* = 0.004**	*z* = 1.99 *p* = 0.047	***z* = 2.5** ***p* = 0.013**	***z* = 3.09** ***p* = 0.002**
	**χ^**2**^(3) = 10.83, *p* = 0.013**					
S3: …my arm felt longer than usual	**S–D:** -0.25 (0.42) **A–D:** -0.9 (0.35) **S–ND:** -1.6 (0.29) **A–ND:** -1.2 (0.3)	*z* = 0.36 *p* = 0.721	***z* = 2.35** ***p* = 0.019**	*z* = 2.07 *p* = 0.038	***z* = 2.7** ***p* = 0.007**	*z* = 1.94 *p* = 0.052
	**χ^**2**^(3) = 11.89, *p* = 0.008**					
S4: …my arm felt shorter than usual	**S–D:** -0.85 (0.34) **A–D:** -0.7 (0.36) **S–ND:** -1.05 (0.35) **A–ND:** -1.0 (0.3)	*z* = 0.45 *p* = 0.651	*z* = 1.12 *p* = 0.265	*z* = 0.5 *p* = 0.62	*z* = 0.84 *p* = 0.4	*z* = 0.81 *p* = 0.42
	χ^2^(3) = 2.02, *p =* 0.568					
S5: …my own arm was out of my control	**S–D:** -1.5 (0.38) **A–D:** -0.4 (0.44) **S–ND:** -1.6 (0.26) **A–ND:** -0.25 (0.38)	***z* = 2.55** ***p* = 0.011**	*z* = 0.17 *p* = 0.862	*z* = 1.94 *p* = 0.052	*z* = 0.32 *p* = 0.75	*z* = 2.37 *p* = 0.018
	**χ^**2**^(3) = 10.07, *p* = 0.018**					
S6: …I couldn’t remember how long my arm was	**S–D:** -0.15 (0.39) **A–D:** -0.45 (44) **S–ND:** -1.45 (0.37) **A–ND:** -0.9 (0.31)	*z* = 0.36 *p* = 0.722	***z* = 2.76** ***p* = 0.006**	*z* = 0.89 *p* = 0.37	***z* = 2.97** ***p* = 0.003**	*z* = 1.94 *p* = 0.052
	**χ^**2**^(3) = 12.29, *p* = 0.006**					
S7: … I couldn’t really tell where my hand was	**S–D:**.1 (0.38) **A–D:**.2 (0.42) **S–ND:** -1.4 (0.36) **A–ND:** -1.0 (0.4)	*z* = 0.73 *p* = 0.467	***z* = 3.35** ***p* = 0.001**	*z* = 0.32 *p* = 0.75	***z* = 2.09** ***p* = 0.002**	*z* = 2.1 *p* = 0.036
	**χ^**2**^(3) = 16.83, *p* = 0.001**					
S8: …the experience of my arm was less vivid than normal	**S–D:** -0.25 (0.38) **A–D:** 0.15 (0.42) **S–ND:** -0.2 (0.42) **A–ND:** 0.25 (0.4)	*z* = 1.01 *p* = 0.315	*z* = 0.31 *p* = 0.759	*z* = 0.92 *p* = 0.36	*z* = 0.11 *p* = 0.91	*z* = 0.89 *p* = 0.37
	χ^2^(3) = 2.46, *p =* 0.482					

Participants rated their level of agreement with the statements using a 7-item Likert scale (i.e., -3 to +3). Results from Friedman tests comparing all conditions are presented in the second column. In addition, in the other columns planned pairwise comparisons with correction for multiple comparisons (α is indicated in the column header) are presented. Significant differences between conditions are marked in **bold** font. Differences between conditions indicate changes as a result of the auditory manipulation. S–D, Synchronous – Displacement; S–ND, Synchronous – No Displacement; A–D, Asynchronous – Displacement; A–ND, Asynchronous – No Displacement.

In order to explore these significant differences and to validate our manipulations of synchronicity and kinaesthetic cues, we looked separately at the differences due to ‘audio-tactile synchronicity’ and due to ‘arm displacement’, by running Wilcoxon Signed Ranks Tests (with correction for multiple comparisons α = 0.025). First, we compared the average of the two Synchronous conditions and of the two Asynchronous conditions. We found that while participants in the Synchronous audio-tactile conditions felt that the sound was caused by them (S1), they did not feel the same for the Asynchronous conditions, thus providing evidence that our manipulation of synchronicity had the expected effect on agency. In addition, participants significantly disagreed more when enquired about the loss of control over their arm (S5) during the Synchronous than the Asynchronous conditions. This less experienced control over the sounds and over hand movement during the Asynchronous conditions provides evidence that our manipulation of synchronicity had the expected effect on agency.

Second, we compared the average of the two Displacement conditions and of the two No Displacement conditions. We found a significant difference between the Displacement and No Displacement conditions in the felt sensation that the sound came from the same location where the hand was (S2). This provides evidence that our manipulation of kinaesthetic cues derived from arm Displacement had the expected effect on the feelings that sound and tap originate at the same location. Importantly, the felt sensation that the sound comes from the same location where the hand is has been previously identified as being fundamental to the auditory-induced changes in perceived tactile distance (Tajadura-Jiménez et al., 2012). We also found that in the Displacement conditions people felt more as the agents of the sounds (S1). Importantly, we found that in the Displacement conditions people reported not being able to tell where one’s hand was (S7), as well as less disagreement with the statements “*my arm felt longer than usual*” (S3) and “*I couldn’t remember how long my arm was*” (S6).

Furthermore, having identified the Synchronous – Displacement condition as the critical condition for which changes in the experience of one’s arm as a result of the auditory manipulation were observed, *post hoc* analyses compared the mean responses to each statement of the questionnaire for Synchronous – Displacement to the responses given after exposure to the other three conditions (with correction for multiple comparisons α = 0.017; see **Table 2**). These analyses further confirmed a difference between our critical Synchronous – Displacement condition and the Asynchronous conditions in the felt sensation of being the agent of the sounds (S1), and between the Synchronous – Displacement condition and the No Displacement conditions, in the felt sensation that the sound came from the same location where the hand was (S2). Importantly, the Synchronous – Displacement condition significantly differed from the Synchronous – No Displacement condition in the sensation that one’s arm felt longer than usual (S3), that one couldn’t remember how long one’s arm was (S6) and that one couldn’t really tell where one’s hand was (S7). This provides evidence that the combination of synchronicity, which resulted in the subjective experience of being the agent of the tapping sounds, and arm displacement, which involved additional kinaesthetic cues signaling a change in hand position, resulted in subjective changes in the perceived length of the arm and in the perceived location of the hand.

#### Summary Experiment 1

These results demonstrate that hearing the tapping sounds with double auditory distance under certain conditions results in a significant change in participants’ perceived tactile distance on the test arm and in the subjective feelings of arm length. First of all, synchrony between the sounds and the actual taps is critical for this change to occur, because it preserves the subjective experience of being the agent of the sounds, as previously indicated in Tajadura-Jiménez et al. (2012). In addition, kinaesthetic cues signaling arm displacement when displacement of the sound source occurs are necessary in order to observe audio-tactile adaptation, as changes were observed for the Synchronous – Displacement but not for the Synchronous – No Displacement condition, while in both conditions the feelings of producing the sound were preserved.

The results on perceived tactile distance were further confirmed by the subjective reports, which show, for the first time, a significant effect on the subjective experience of arm length (S3). Importantly, we observed significant differences between the Synchronous – Displacement and the Synchronous – No Displacement conditions in the felt sensations that the sound came from the same location where the hand was (S2), and that one could not really tell where one’s hand (S7) was. It exists the possibility that these differences derive from an effect of the posture adopted by participants in the No Displacement conditions. In particular, we identified two differences between Displacement and No Displacement conditions due to posture, which did not allow us to conclude that the difference in results between these conditions was only due to the presence/absence of kinaesthetic cues signaling arm displacement. First, the distance between sound source and hand was larger in the No Displacement conditions (it increased from 25 to 125 cm, as the hand is kept at 25 cm but the sound source moves from a position 50 cm away to a position 150 cm away) than in the Displacement conditions (where it increased from 25 to 75 cm, as the hand moves from a position 25 cm away to a position 75 cm away, and the sound source moves from a position 50 cm away to a position 150 cm away). Could this smaller hand-sound source distance in the Displacement conditions have accounted for the difference in felt sensation that the sound came from the same location where the hand was (S2)? Second, the distance between hand and body torso differed between conditions. While in the No Displacement conditions the hand was kept 25 cm away, in the Displacement condition the hand could be as far as 75 cm away. Could this larger hand-body torso distance in the Displacement conditions have accounted for the difference in felt sensation that one could not really tell where one’s hand was (S7)?

Given these findings and the discussed possible confounds, a second experiment was run. Experiment 2 served to control for the possible confounding variables by having a modified version of the No Displacement conditions in which participants kept their arm stretched and their hand placed at the last tapping-position, thus far away from the participants’ torso. With this modified version of the No Displacement conditions, we made sure, first, that in both the Displacement and the No Displacement conditions the initial distance between the sound source and hand location was 25 cm, and that the maximum distance was 75 cm. Second, we made sure that the hand location in the No Displacement condition equalled the maximum hand-body torso distance in the Displacement condition, this is, 75 cm.

Hence, Experiment 2 was run with the hypothesis that if different results were found between the Displacement and No Displacement conditions, we could conclude that they were due to the presence of kinaesthetic cues signaling arm displacement, and not due to differences in the maximum hand-sound source, and hand-body torso distances.

## Experiment 2

### Materials and Methods

#### Participants

Seventeen participants took part in the experiment. We applied the same participant selection criteria as in Experiment 1, and the experiment was conducted in accordance with the same ethical standards. Three participants were removed from the analyses, given that two of them were unable to complete the audio-tactile “tapping” task as required, due to difficulties in remembering the instructions, and one was unable to complete the “tactile distance” task, due to lack of tactile sensitivity in the arm. Therefore, only results from fourteen participants are reported here (*M*_age_ ± SD = 23.64 ± 3.6 years; age range from 18 to 30 years; seven females).

#### Apparatus, Materials, Procedure, and Data Analyses

Identical apparatus, materials, and data analyses to the ones in Experiment 1 were used. The procedure used was also identical to the one in Experiment 1, except that in this case, during the audio-tactile “tapping” task in the No Displacement trials, participants were required to keep their right arm stretched and tap always at the same, sixth tapping-position.

### Results

#### Behavioral Results

The mean PSE values ± SE are presented in **Table 3**. Identical analyses to those in Experiment 1 were conducted and the behavioral results mirrored those in Experiment 1. After validating the Pre-test values as baseline, and the treatment of both post-tests as a single test, our main analysis focused, as before, on the effect of audio-tactile stimulation across conditions.

**Table 3:** Results from Experiment 2.

Time of test	Synchronous – Displacement	Asynchronous – Displacement	Synchronous – No Displacement	Asynchronous – No Displacement
Pre-test	1.008 (0.032)	1.033 (0.025)	1.079 (0.046)	1.009 (0.032)
Post-test	1.127 (0.044)	1.052 (0.036)	1.101 (0.05)	1.093 (0.045)

Mean PSE ± SE for each experimental condition and for both the Pre- and Post-test.

**FIGURE 3:**
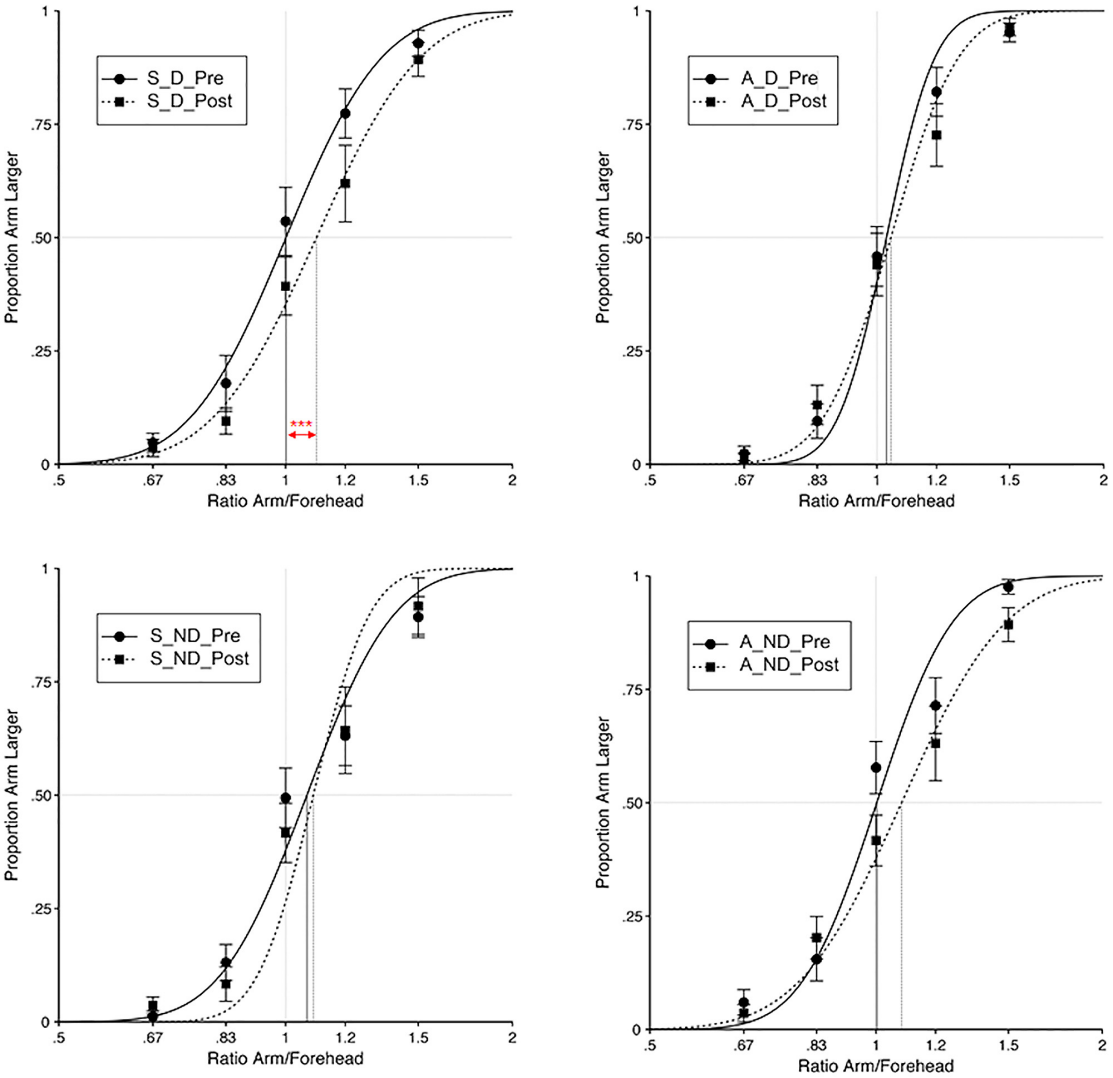
**Results from Experiment 2.** For each experimental condition (S–D, Synchronous – Displacement; S–ND, Synchronous – No Displacement; A–D, Asynchronous – Displacement; A–ND, Asynchronous – No Displacement), and for both the Pre- and Post-test, the proportion of judgements that the distance between dual tactile stimuli on the right arm felt greater than on the forehead was analyzed as a function of the ratio of the length of the arm and forehead stimuli (i.e., 4/6, 5/6, 1, 6/5, or 6/4). Curves are cumulative Gaussian function fits to the group data, for each condition, with least-squared regression. Error bars indicate the SEM. Vertical lines indicate the interpolated PSE between the perceived distance on the arm and on the forehead. Red asterisks denote a significant change in PSE from Pre- to Post-test (^∗∗∗^denotes *p* < 0.001, corrected for multiple comparisons). Note that an increase in the PSE meant that perceived tactile distances on the arm were felt smaller, as compared to distances on a reference location.

A normality check of the residuals with Shapiro–Wilk tests and Q–Q plots showed moderate deviations from normality for two out of the eight variables (Synchronous – No Displacement pre-test: *W*(14) = 0.83; *p* = 0.015; Synchronous – No Displacement post-test: *W*(14) = 0.83; *p* = 0.015), and hence we opted for the use of ANOVAs. As in Experiment 1, the 3-way interaction ‘audio-tactile synchronicity’ by ‘arm displacement’ by ‘time of test’ was significant [*F*(1,13) = 7.04; *p* = 0.02], as well as the main effect of ‘time of test’ [*F*(1,13) = 14.01; *p* = 0.002], while the other main effects or interactions failed to reach significance (all *p*s > 0.05; see **Figure 3**).

In order to explore the 3-way interaction, we conducted two further 2 × 2 within-subjects ANOVA, one for the Displacement and one for the No Displacement condition, with factors ‘audio-tactile synchronicity’ (Synchronous and Asynchronous) and ‘time of test’ (Pre-test and Post-test). The ANOVA for the Displacement condition revealed a significant main effect of ‘time of test’ [*F*(1,13) = 14.28; *p* = 0.002], as well as a significant 2-way interaction ‘audio-tactile synchronicity’ by ‘time of test’ [*F*(1,13) = 9.47; *p* = 0.009], while the main effect of ‘audio-tactile synchronicity’ was not significant (*p* > 0.05). Independent-samples *t*-tests showed that the observed interaction was driven by a significant increase in the PSE (i.e., perceived tactile distances on the arm felt smaller) from Pre- to Post-test in the Synchronous – Displacement condition [*t*(13) = -4.40, *p* < 0.001], which was not observed for the Asynchronous – Displacement condition (*p* > 0.05). The ANOVA for the No Displacement condition yielded a significant main effect of ‘time of test’ [*F*(1,13) = 4.65; *p* = 0.05], while the main effect of ‘audio-tactile synchronicity’ or its interaction with ‘time of test’ were not significant (all *p*s > 0.05).

#### Subjective Results

The full set of statements, mean responses and test for significance are presented in **Table 4**. Identical analyses to those in Experiment 1 were conducted and the subjective results mostly mirrored those in Experiment 1. We observed significant differences between the four conditions for statements S1, S2, S3, and S5.

**Table 4:** Mean ratings (±SE) and tests for significance for each questionnaire item across conditions in Experiment 2.

During the audio-tactile stimulation it seemed like…	Mean ratings (±SE) and results of Friedman test	Synchronous vs. Asynchronous (α = 0.025)	Displacement vs. No Displacement (α = 0.025)	S–D vs. A–D (α = 0.017)	S–D vs. S–ND (α = 0.017)	S–D vs. A–ND (α = 0.017)
S1: …the sound I heard was caused by me	**S–D:** 2.07 (0.37) **A–D:** 0.07 (0.67) **S–ND:** 2.21 (0.35) **A–ND:** -0.71 (0.59)	***z* = 3.08** ***p* = 0.002**	*z* = 1.44 *p =* 0.149	***z* = 2.46** ***p* = 0.014**	*z* = 0.26 *p* = 0.79	***z* = 3.00** ***p* = 0.003**
	**χ^**2**^(3) = 19.8, *p* < 0.001**					
S2: …my hand was at the same location as the sound	**S–D:** 1.71 (0.41) **A–D:** 0.5 (0.51) **S–ND:** -0.29 (0.61) **A–ND:** -1.36 (0.44)	***z* = 2.56** ***p* = 0.01**	*z* = 2.77 ***p* = 0.006**	*z* = 1.7 *p* = 0.089	*z* = 2.25 *p* = 0.025	***z* = 2.96** ***p* = 0.003**
	**χ^**2**^(3) = 12.32, *p* = 0.006**					
S3: …my arm felt longer than usual	**S–D:** -0.86 (0.36) **A–D:** -1.29 (0.34) **S–ND:** 0.07 (0.42) **A–ND:** -0.93 (0.41)	***z* = 2.39** ***p* = 0.017**	*z* = 1.84 *p* = 0.065	*z* = 1.51 *p* = 0.13	*z* = 1.98 *p* = 0.048	*z* = 0.69 *p* = 0.49
	**χ^**2**^(3) = 10.84, *p* = 0.013**					
S4: …my arm felt shorter than usual	**S–D:** -0.71 (0.38) **A–D:** -1.0 (0.38) **S–ND:** -1.36 (0.36) **A–ND:** -0.79 (0.38)	*z* = 0.79 *p* = 0.429	*z* = 0.88 *p* = 0.38	*z* = 1.19 *p* = 0.23	*z* = 2.04 *p* = 0.041	*z* = 0.32 *p* = 0.75
	χ^2^(3) = 6.2, *p =* 0.102					
S5: …my own arm was out of my control	**S–D:** -1.79 (0.39) **A–D:** -0.36 (0.44) **S–ND:** -1.29 (0.40) **A–ND:** -0.71 (0.50)	*z* = 2.21 *p* = 0.027	*z* = 0.36 *p* = 0.720	*z* = 2.39 *p* = 0.017	*z* = 1.22 *p* = 0.22	*z* = 1.98 *p* = 0.048
	**χ^**2**^(3) = 11.75, *p* = 0.008**					
S6: …I couldn’t remember how long my arm was	**S–D:** -1.07 (0.43) **A–D:** -0.93 (43) **S–ND:** -0.36 (0.37) **A–ND:** -0.93 (0.46)	*z* = 0.78 *p* = 0.436	*z* = 1.45 *p* = 0.146	*z* = 0.71 *p* = 0.48	*z* = 1.91 *p* = 0.056	*z* = 0.00 *p* = 1.00
	χ^2^(3) = 2.77, *p* = 0.428					
S7: … I couldn’t really tell where my hand was	**S–D:** -1.14 (0.40) **A–D:** -0.21 (0.42) **S–ND:** -0.64 (0.32) **A–ND:** -1.0 (0.44)	*z* = 1.09 *p* = 0.278	*z* = 0.57 *p* = 0.57	*z* = 1.80 *p* = 0.072	*z* = 0.90 *p* = 0.365	*z* = 0.00 *p* = 1.00
	χ^2^(3) = 4.18, *p* = 0.243					
S8: …the experience of my arm was less vivid than normal	**S–D:** -0.86 (0.40) **A–D:** -0.21 (0.41) **S–ND:** -0.43 (0.23) **A–ND:** -0.14 (0.49)	*z* = 1.5 *p* = 0.134	*z* = 1.33 *p* = 0.185	*z* = 1.26 *p* = 0.21	*z* = 0.84 *p* = 0.40	*z* = 2.16 *p* = 0.031
	χ^2^(3) = 3.41, *p* = 0.332					

Participants rated their level of agreement with the statements using a 7-item Likert scale (i.e., -3 to +3). Results from Friedman tests comparing all conditions are presented in the second column. In addition, in the other columns planned pairwise comparisons with correction for multiple comparisons (α is indicated in the column header) are presented. Significant differences between conditions are marked in **bold** font. Differences between conditions indicate changes as a result of the auditory manipulation. S–D, Synchronous – Displacement; S–ND, Synchronous – No Displacement; A–D, Asynchronous – Displacement; A–ND, Asynchronous – No Displacement.

When comparing the average of the two Synchronous conditions and of the two Asynchronous conditions, apart from the effects reported in Experiment 1 on feelings of being the agent of the sound (S1) and of one’s arm being out of one’s control (S5), we also found that in the Synchronous conditions people felt more that the sound came from the same location where the hand was (S2), and disagreed less with the statement “*my arm felt longer than usual*” (S3). When comparing the average of the two Displacement conditions and of the two No Displacement conditions, we found a similar effect as that reported in Experiment 1 on feelings that the sound came from the same location where one’s hand was (S2). Finally, when comparing the Synchronous – Displacement responses to those responses given after exposure to the other three conditions we found similar effect as those reported in Experiment 1 on feelings of being the agent of the sound (S1) and that the sound came from the same location where the hand was (S2). We also observed a close to significant larger loss of control of one’s own arm (S5) in the Asynchronous conditions as compared to the Synchronous – Displacement condition.

#### Direction of Changes in Represented Arm Length

**FIGURE 4:**
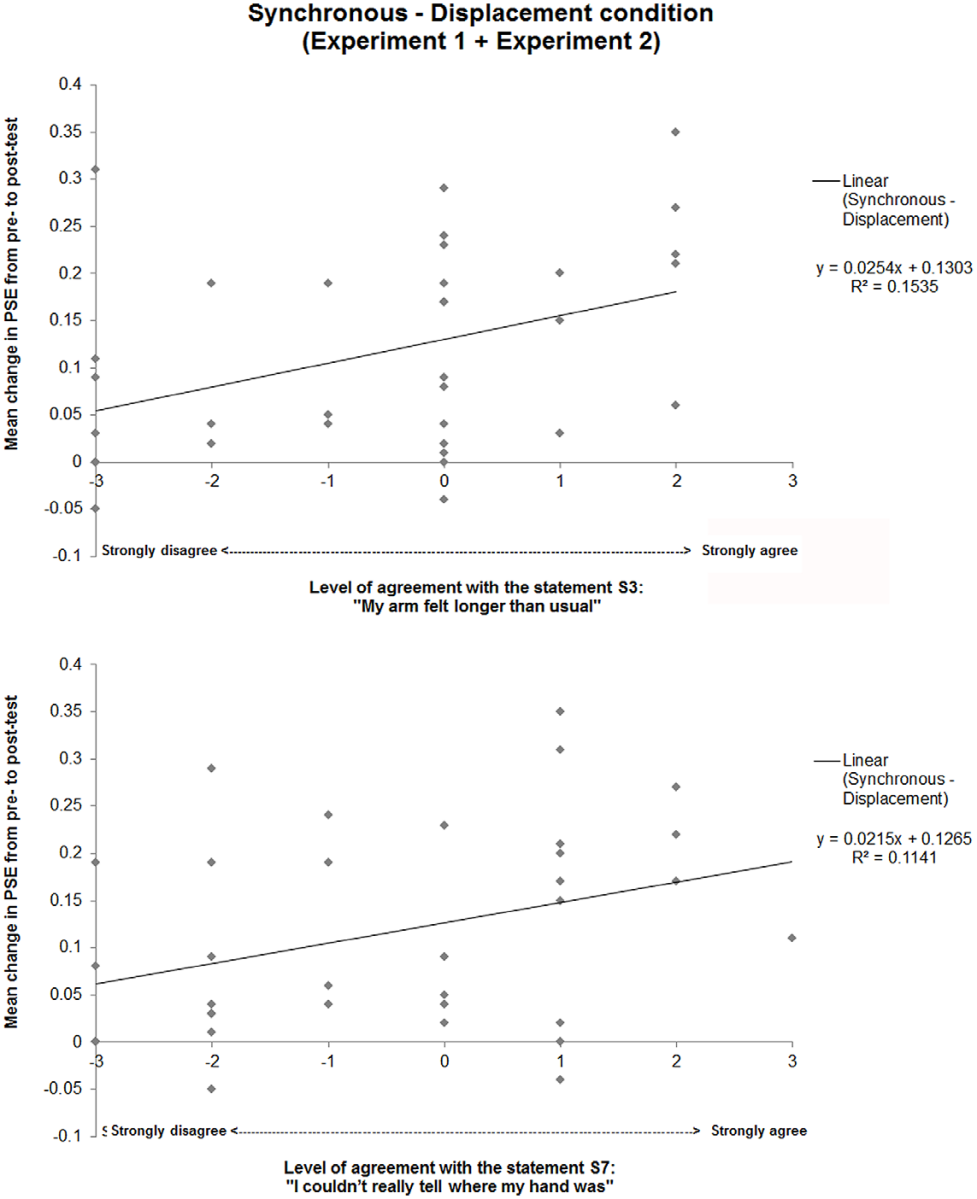
**Correlation between behavioral and subjective results for the Synchronous – Displacement condition (data pooled from both experiments).** There were positive correlations between the self-reported level of agreement with the statements S3 and S7 and the magnitude of change from Pre- to Post-test in the PSE between the perceived tactile distance on the arm and on the forehead. The diagonal lines are linear regression lines; their equations and *R*^2^ values are indicated in the graph.

We investigated how the observed tactile distance changes in the Synchronous – Displacement condition related to participants’ subjective experience of changes in arm length. Given that the Synchronous – Displacement condition was identical in Experiments 1 and 2, we pooled the results from the total 34 participants in both experiments and performed correlation analyses between behavioral and subjective data. In particular, we looked at Spearman’s rho correlations between the change from Pre- to Post-test in PSE in the tactile distance task and the self-reported level of agreement for all statements (S1–S8) in the Synchronous – Displacement condition. Results showed that changes in PSE correlated significantly with changes in level of agreement with the statement S3 “*my arm felt longer than usual*” [*r*_S_(34) = 0.41, *p* = 0.015] and with the statement S7 “*I couldn’t really tell where my hand was*” [*r*_S_(34) = 0.36, *p* = 0.038], while correlations with data from the other statements were all not significant. In particular, linear regression analyses revealed that positive changes in PSE predicted increased feelings of one’s arm being longer than usual and of not being able to tell where one’s hand was (see **Figure 4**).

#### Summary Experiments 1 and 2

In Experiment 2 we controlled for the effect of arm posture adopted in the conditions lacking kinaesthetic cues signaling arm displacement (i.e., No Displacement conditions). In particular, we made sure that the lack of results for the No Displacement conditions in Experiment 1 was independent of the distance between body torso and hand (hand close to the body torso in Experiment 1 and far from the body torso in Experiment 2), and of the distance between hand and sound source, which was larger for the posture adopted in Experiment 1 than for the posture in Experiment 2.

The obtained results support the finding that exposure to the tapping sounds with double auditory distance significantly changes participants’ perceived tactile distance on the test arm, in comparison to the reference location. Importantly, Experiment 1 and Experiment 2, together demonstrate that both synchrony between the tapping sounds and the actual taps of participants, and the update in kinaesthetic cues during the displacement of one’s arm while tapping, are critical conditions for this change to occur. In other words, changes in perceived tactile distance on the arm do not occur in the absence of kinaesthetic cues signaling arm displacement (i.e., when the arm remained tapping at a fixed location), even when the feelings of one being the agent of the sounds are preserved. In addition, results from both experiments suggest that changes in perceived tactile distance on the arm correlate with feelings of one’s arm changing length.

## Discussion

Taken together our results elucidate necessary factors for auditory-induced recalibration of perceived tactile distances to occur. Extending previous results (Tajadura-Jiménez et al., 2012), we show that the manipulation of the auditory distance of the triggered tapping sounds can change the perceived tactile distance on the arm used for tapping. Importantly, we here show that the involvement of kinaesthetic cues signaling arm displacement during tapping when displacement of the sound sources occurs is a necessary condition for these sound sources to induce changes in the perceived tactile length of external objects. In particular, the feeling of being the agent of the tapping sounds is not sufficient to induce changes in the perceived tactile distance, but a coherent representation of the motor command sent to the displacing and tapping arm and the sound feedback received from the tapping action needs to arise in order to observe such changes. Furthermore, we provide the first evidence that self-produced sounds can evoke consciously perceived changes in body-representation, specifically in the represented arm length, and that these changes correlate with changes in the perceived tactile length of external objects in contact with one’s arm. In the following sections, we discuss the implications of the observed effects and the limitations of the study.

### Is the Feeling of Being the Agent of the Sound Sufficient to Change the Perceived Tactile Distance?

Our study shows that the feeling of being an agent of the sound, achieved by keeping temporal contingency between the action and its attributed sound, alone is not sufficient to observe auditory-induced changes in perceived tactile distance. Instead, the involvement of kinaesthetic cues signaling arm displacement when displacement of the sound sources occurs is also necessary for these changes to occur, provided that the distance at which sounds originate is within certain limits from the tapping hand (i.e., we observed changes after exposure to tapping sounds originating at double but not at quadruple the distance at which participants actually tapped; Tajadura-Jiménez et al., 2012).

It should be noted that since people made voluntary movements when tapping throughout all experimental conditions, they did retain a basic sense of agency in so far they themselves were moving. However, our manipulation of temporal contingency impacted on the sense of agency, as it influenced the experience of being the agent of the sounds and of being in control over hand movement, as evidenced by the subjective results. It should also be noted that, while an overall increase in PSE from Pre- to Post-test occurred in all conditions, such systematic baseline shifts after adaptation are often reported in multisensory adaptation paradigms. For instance, exposure to fixed audiovisual time lags for several minutes results in shifts in subjective simultaneity responses in the direction of the exposure lag, indicating a perceptual temporal recalibration of multisensory perception (Fujisaki et al., 2004; Vroomen et al., 2004). While we cannot fully clarify here whether the baseline change observed in our experiments derives from some sort of perceptual temporal recalibration of multisensory perception or other processes, what is critical in our results is that this change in PSE from Pre- to Post-test significantly interacted with synchronicity (i.e., with the presence/absence of feelings of agency), in the Displacement conditions.

We suggest that these findings can be interpreted in the context of the proposed ‘forward internal models’ of motor-to-sensory transformations (Wolpert and Ghahramani, 2000). These models serve to predict the movement dynamics and the sensory outputs that derive from one’s actions (i.e., reafference). Hence, when we move an arm, the central nervous system estimates the next state (e.g., the next position of the hand) by combining the current efferent motor outflow (the motor commands sent to the arm) with the predictions of arm’s dynamics for the current state. The central nervous system also estimates the sensory reafference that will accompany the next state by combining the current reafferent multisensory inflow with the sensory predictions for the current state. The discrepancies between prediction and reafference are used to do adjustments in next state estimates (Wolpert et al., 1995), as well as to do fine adjustments in the subsequent motor commands (Blakemore et al., 2002). Studies introducing temporal and spatial discrepancies between movement and its visual consequences have shown that only discrepancies between prediction and reafference exceeding a certain threshold become available to awareness (Blakemore et al., 2002). Trespassing this threshold can result in delusions of control over produced actions, although the exact threshold for these discrepancies to reach awareness is debated. Indeed, this threshold can be relatively large, as long as our intentions are successfully achieved (Blakemore et al., 2002).

In our study, which involves motor-to-sensory transformations when moving an arm, we observed changes in perceived arm length for the Synchronous – Displacement condition, but not for the other conditions. We suggest that this condition provides a better temporal and spatial match between reafference and sensory predictions than the other conditions. In the Synchronous – Displacement condition, as opposed to the Asynchronous conditions, there is a temporal agreement between the action and its attributed sound, which results in the feeling of being the agent of the sound (e.g., Moore et al., 2009). Nevertheless, we show that the conscious experience of agency alone is not enough to evoke changes in represented arm length, because these changes were not observed in the Synchronous – No Displacement condition. The Synchronous – Displacement condition, in addition to temporal synchrony, provides a better spatial match between reafference and sensory predictions made on the basis of the efferent motor outflow: the kinaesthetic cues in the motor outflow indicate a change in location of the hand and, similarly, the reafferent sensory input indicates a change in location of the sound source in the same direction. It is important to note that in the Synchronous – Displacement condition the feeling that “*the sound comes from the same location where the hand is*” (see S2) is preserved, even if the tapping sounds originated at double the distance at which participants actually tapped. This temporal and spatial mismatch reduction during the action-perception loop allows forming an association between action and sound (Ernst and Bülthoff, 2004). It should also be noted that our design indirectly includes also the testing of a condition where neither kinaesthetic cues signal a change in location of the hand nor the reafferent sensory input indicates a change in location of the sound source. The last ten taps of the Synchronous – No Displacement condition in Experiment 2 correspond to a situation in which participants do not displace their arm and sound sources are at double the distance to the tapping location. While this exposure is short (∼10 s), work on other sensory-driven bodily illusions have shown that such short periods may be enough to elicit the illusions (e.g., Ehrsson et al., 2004). However, we did not observe any significant behavioral or subjective changes for this condition. These results seems to suggest that kinaesthetic cues signaling arm displacement are needed for recalibrating arm length in this context, at least for short-term exposures. The testing of long-term exposure remains beyond the scope of this study, but it is nevertheless a topic interesting for further research.

Taken in this context of ‘forward internal models’, our results add to the theories on these models. These theories have mainly considered that the reafferent sensory inflow used by forward models is constituted by visual and proprioceptive information (Wolpert et al., 1995). Here we propose, not only that action sounds also constitute part of this reafferent inflow, as suggested by recent neuroimaging studies demonstrating the link between action sounds and brain areas involved in the planning, preparation, and observation of actions involved in the production of those sounds (for a review see Aglioti and Pazzaglia, 2010), but also that predictions related to action sounds must fit with kinesthetic cues related to the performed actions in order to make use of the auditory inputs to update the model.

Furthermore, our study sheds light into the magnitude of the threshold for which the model can compensate for auditory-motor spatial discrepancies. We showed that action sounds may be attributed to the outputs of the actions performed by one’s hand even when the sounds originate at double the distance at which the hand is, provided that the feelings of agency and kinaesthetic cues signaling arm displacement are preserved. Previously we also showed that when the sound originates at quadruple the distance auditory-motor spatial discrepancies become too large (Tajadura-Jiménez et al., 2012). This threshold is similar to the one reported in a related study in the visual domain, in which the illusion of owning a very long arm, seen from a first-person perspective, starts breaking when the length of this arm exceeds three times the actual length of the participants’ arm (Kilteni et al., 2012). While future work should further clarify the exact threshold for temporal and spatial discrepancies to disrupt these illusions, we hypothesize that this threshold may relate to sources fallings inside the represented near space.

Finally, theories of ‘forward internal models’ have mainly discussed how these models are continuously updated by sensorimotor information in order to estimate, for instance, the position and velocity of a hand moving (Wolpert et al., 1995), as well as to do fine adjustments in the subsequent motor commands (Blakemore et al., 2002). Our study provides more insight into the updating of these models, by showing that the auditory feedback on one’s hand actions is not only used to update the estimated position of the hand, but also to update the represented arm length that allows the hand to be in that position. We suggest that when engaged in limb actions, in order to estimate the current position of the hand, predictions must integrate, apart from multimodal information extracted from previous sensorimotor feedback, internal knowledge about the configuration and length of the limbs (i.e., mental representation of one’s limbs). Hence, when moving the limb the sensory feedback is weighted against the predictions and, if potential discrepancies arise but are kept below a certain threshold, these discrepancies are used to do fine adjustments in the mental representation of one’s body. In support of our suggestion, previous studies have shown that the representation of an action engages a mental representation of the general body structure that allows this action to be produced (Holmes and Spence, 2004; Maravita and Iriki, 2004). Indeed, studies on audio–motor mirroring of action sounds have shown that action representation engages both agency and mental representations of the body part involved in the action (see Aglioti and Pazzaglia, 2010). Because our body configuration can change, the internal body-representation is plastic to adapt to changing circumstances by tuning to the incoming sensory feedback. Importantly, as mentioned above, we showed that predictions related to action sounds must fit with kinesthetic cues related to the performed actions in order to make use of the auditory inputs to change body-representation.

### Do Action Sounds Change the Subjective Experience of Arm Length?

We here show for the first time that action sounds can indeed change the subjective experience of arm length. Our results demonstrate that the level of agreement with statement S3 “*my arm felt longer than usual*” significantly changed across conditions. Moreover, for the critical Synchronous – Displacement condition, we observed that those participants showing larger levels of agreement with statement S3 showed larger audio-tactile driven increases in PSE for the tactile distance judgment task, thus suggesting that changes in perceived tactile distance relate to experienced arm elongation.

It should be noted that in our previous study, the observed behavioral changes in represented arm length were not accompanied by significant changes in the subjective experience of arm length, thus providing evidence that changes in body-representation can occur outside of awareness (Holmes and Spence, 2004; Maravita and Iriki, 2004). We argue that the listening experience provided by the headphone-based setup may have been a factor favoring that changes in represented arm length reached awareness. Note that in the current study we simulated the array of auditory spatial positions, which allowed using headphones to present the tapping sounds, instead of loudspeakers, which were used in our previous study (Tajadura-Jiménez et al., 2012). Although we did not directly measure immersion, headphone-based listening has previously been shown to provide more intense, immersive experiences, as compared to loudspeaker-based listening (Kallinen and Ravaja, 2007; Tajadura-Jiménez et al., 2008, 2011). Moreover, in the current setup participants were blindfolded. Therefore, visual cues, as well as auditory cues other than the experimental stimuli, were reduced. These differences might have favored immersion on the listening experience and positively impacted on the subjective experience of arm length.

### Direction of Changes in Perceived Tactile Distance

Recently, there has been some controversy on the direction of changes resulting from the tactile-distance task. Studies on the effects on perceived tactile distance of body-related inputs from sensory modalities other than sound have shown that an increase in the represented part of the body relates either to an increase (e.g., Taylor-Clarke et al., 2004; de Vignemont et al., 2005; Lopez et al., 2012) or to a decrease (Canzoneri et al., 2013a,b) in perceived tactile distance on that part of the body. Our present results add to this controversy. Here we demonstrate that exposure to manipulated auditory body-related inputs results in the perceived tactile distances on the arm being felt smaller, as compared to distances on a reference location. However, in our previous study (Tajadura-Jiménez et al., 2012) exposure to similar inputs resulted on tactile distances felt bigger.

It should be noted that the fact that tactile distances were felt smaller on the arm is not necessarily in contrast with an increase in the represented length of the arm. Indeed, we showed that larger feelings of arm elongation correlated with smaller felt tactile distances. In the studies by Canzoneri et al. (2013a,b), other additional behavioral measures supported the interpretation that the decrease in perceived tactile distance in the arm following tool-use results from an increase in the represented length of the arm. These authors related their findings to other studies showing that the larger one’s body (or body part) is perceived, the smaller objects external to one’s body are perceived (Linkenauger et al., 2011; van der Hoort et al., 2011). It has been suggested that one’s body is used as a “perceptual ruler” to measure object’s size (Linkenauger et al., 2011; Canzoneri et al., 2013b). This controversy on the direction of changes resulting from the tactile-distance task has been recently discussed in a publication by Miller et al. (2014). They nicely summarize the two opposing views in previous studies, in favor of either an inverse or a proportional relationship between represented body size and perceived tactile size, and they suggest that none of the views is correct or incorrect, but rather that one needs to take into account possible factors that might influence how tactile information is used when providing the tactile distance judgements.

Tactile distance judgements for stimuli delivered on the arm are both dependent upon the mental representation of arm length, as well as upon the geometry of receptive fields (RFs) in primary somatosensory cortex (SI; Longo and Haggard, 2011). Miller et al. (2014) discuss that visual bodily feedback can result in an update in the stored visual body template and cause reorganization of SI RF geometry (Haggard et al., 2007). They suggest that top–down sensory signals can cause this reorganization of SI RF geometry leading to changes in tactile size perception. Similarly, we previously suggested that tactile perception is referenced to an implicit body-representation which is updated through auditory feedback, presumably by auditory-induced recalibration of SI RFs (Tajadura-Jiménez et al., 2012). However, both Miller et al. (2014) and us suggest that, in addition to reorganization of SI RF geometry, there might be other top–down factors (e.g., contextual/task demands) that might influence the direction of the tactile distance judgments: tactile information is used differently in distinct, but related tactile tasks, such as tactile distance perception and tactile localization, which are both affected by sensory information on body size, presumably following reorganization of SI RF geometry.

We suggest that task differences between our two studies may explain why this opposite direction of the results is observed. We introduced differences in the task in order to use a more sound methodology by addressing some potential biases affecting previous results. A first difference between the two studies is the body location used as reference: while previously we used as reference location the left arm, here we used the forehead. Previous studies have shown asymmetries in perceived arm length (i.e., participants may perceive their right arm to be longer than their left arm), which correlate with factors such as participants’ handness and hand strength (Linkenauger et al., 2011). It might be that these asymmetries are also affected by the experimental task, and in order to control for this, we chose as reference location the forehead, a location that has been previously used in a number of studies assessing changes in perceived body size (de Vignemont et al., 2005; Lopez et al., 2012; as well as the studies by Canzoneri et al., 2013a,b). Second, while previously the task for participants was to indicate whether the distance on the left or on the right arm felt greater, here they had to indicate whether the two points felt farther apart in the first or the second stimulated location. This change in task was introduced because our previous task may suffer from a first-order response bias, while the current task does not (Longo and Haggard, 2011). The current task has been previously used in other studies assessing tactile size perception (Longo and Haggard, 2011). Third, while previously the minimum experienced tactile distance was 2 cm, here it was 4 cm. This change was introduced in order to make sure that tactile distances were clearly suprathreshold at both body locations (Nolan, 1982, 1985), following the suggestion made by Canzoneri et al. (2013a,b).

To sum up, in our view, the nature of the task and the specific body parts used as test and reference locations seem to play a role in this relationship between tactile distance judgements and represented arm length changes, as different reference frames may be used for different body parts. Our study was not designed to directly tease these effects apart and therefore the exact relationship between tactile distance judgments and represented arm length remains open for further research. Having said this, while in our previous study we could not interpret the behavioral results in relation to the direction of changes in the represented arm length because the observed changes in perceived tactile distance were not accompanied by changes in the phenomenal experience of arm length (neither feeling that the arm elongated nor that it shrank was reported), in our present study we did observe such phenomenal changes. Changes in level of agreement with the statement “*my arm felt longer than usual*” significantly correlated with changes in perceived tactile distance on the arm, and in particular, larger increases in Pre- to Post-test PSE for the tactile distance task predicted larger feelings of one’s arm being longer than usual. Given this subjective evidence, we suggest that the observed changes in perceived tactile distance relate to arm elongation.

## Conclusion

Our results show that self-produced tapping sounds can change perceived tactile distances but only when cues indicating both that one is the agent of the sounds and that when sound sources displace the tapping arm also displaces (i.e., kinaesthetic cues) are preserved. The present study adds to theories on forward internal models of motor-to-sensory transformations by showing that predictions related to action sounds must fit with kinesthetic cues related to the performed actions in order to make use of the auditory inputs to change body-representation. These cues reduce the spatial mismatch in the motor-to-sensory transformations, allowing a coherent, and robust sensory percept to emerge. Our results thus provide further insights on the necessary conditions (i.e., synchrony, agency, kinaesthesia) to observe audio-tactile influences on the coherence of body-representations. In addition, we showed, for the first time, that self-produced sounds can evoke consciously perceived changes in body-representation if a sufficiently immersing setup is provided. Finally, our results suggest that the nature of the tactile distance task and the specific body parts involved in the task may influence tactile distance judgements, as they may involve different reference frames. With the task used in this study, a decrease in perceived tactile distance on the arm caused by audio-tactile adaptation seemed to predict feelings of one’s arm elongating.

Future research should determine whether the kinaesthetic feedback should be resulting from active movements or whether passive movement is sufficient in order to observe audio-tactile influences on the coherence of body-representations, as well as determine whether the use of other sounds (i.e., non-action sounds) could have similar influences. Future studies may also test how long the effects of the audio-tactile adaptation last, as well as whether the observed effects would be enhanced or would diminish due to longer audio-tactile adaptation periods. Finally, it remains to be tested whether experienced changes in arm length scale with changes in the extent of the represented near space, as previous research has found a relation between arm lengths and represented near space (Longo and Lourenco, 2007).

This research on the dependency of body-representation upon auditory information complements previous research addressing the contribution of visual, tactile (Botvinick and Cohen, 1998), proprioceptive (de Vignemont et al., 2005; Ehrsson et al., 2005), and vestibular (Lopez et al., 2012; Ferrè et al., 2013) channels to body-representation (for a recent review see Kilteni et al., 2015).

## Author Contributions

All authors contributed to the conception and design of the work, interpretation of data and revision of the drafts of the work. AT acquired and analyzed the data, and drafted the work. All authors agreed to be accountable for all aspects of the work in ensuring that questions related to the accuracy or integrity of any part of the work are appropriately investigated and resolved, and approved this final version of the manuscript.

## Conflict of Interest Statement

The authors declare that the research was conducted in the absence of any commercial or financial relationships that could be construed as a potential conflict of interest.
